# Supplementary material for: Suppressive cancer nonstop extension mutations increase C-terminal hydrophobicity and disrupt evolutionarily conserved amino acid patterns
Source: Nat Commun. 2024 Oct 25;15:9209. doi: 10.1038/s41467-024-52779-4 (PMC11502859; doi:10.1038/s41467-024-52779-4)
Supplement: Supplementary file 1 — Supplementary Information [file 41467_2024_52779_MOESM1_ESM.pdf]

Suppressive cancer nonstop extension mutations increase C-terminal hydrophobicity and disrupt evolutionarily conserved amino acid patterns

Avantika Ghosh, Marisa Riester, Jagriti Pal, Kadri-Ann Lainde, Carla Tangermann, Angela Wanninger, Ursula K. Dueren, Sonam Dhamija and Sven Diederichs

SUPPLEMENTS

**Supplementary Figures S1 - S5 & Legends**	(this file)
**Supplementary Data 1 - 3**	(separate files)
**Source Data File**	(separate file)

Figure S1

(a)

Type of extension	No. of Oligos
Negative Control (No Extension, STOP codon)	3
Extension NSdb_MUT	2335
Extension NSdb_WT	2172
Artificial sequences	200
Known degrons	25
Total	4735

(b)

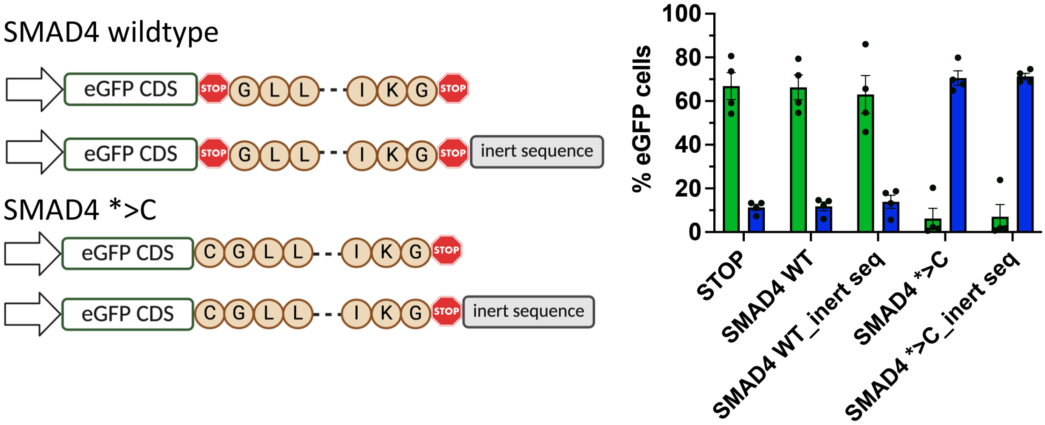

(c)

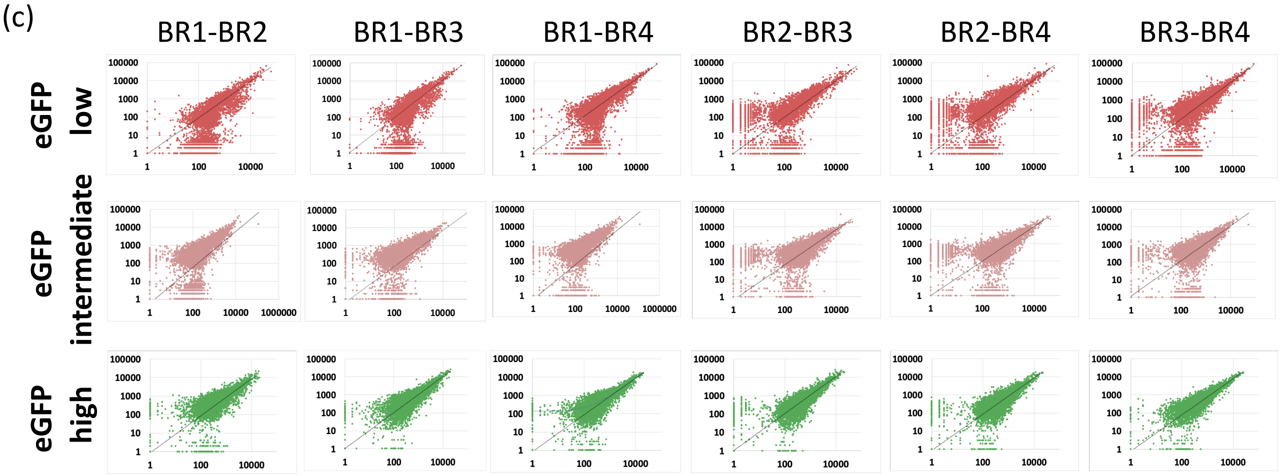

(d)

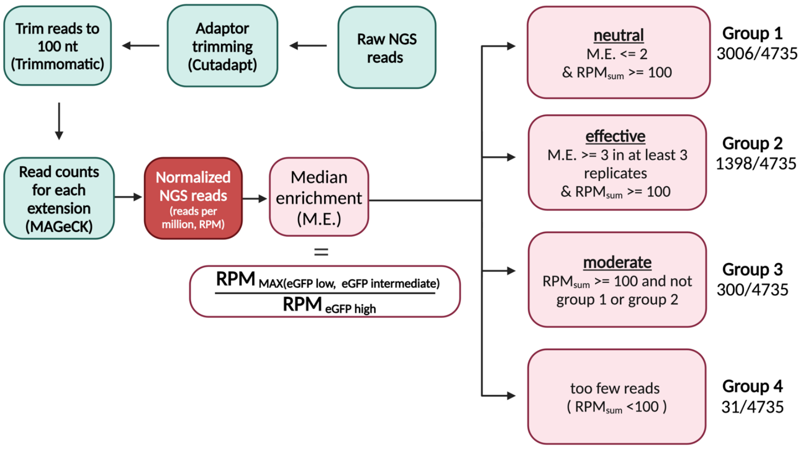

(e)

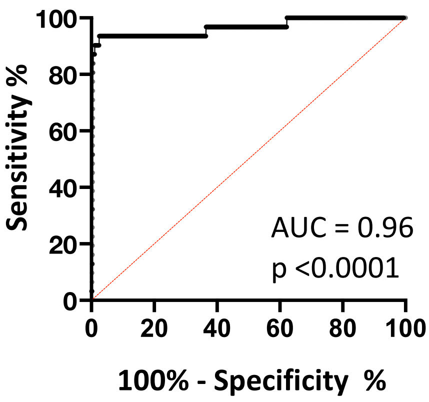

Supplementary Figure S1: Extension library design, quality and analysis for the high-throughput screening platform for testing cancer-derived nonstop extensions

a, Table depicting the various types of extensions in the high-throughput screen along with the corresponding number of oligos for each type with three STOP codons as negative controls, 25 known degrons as positive controls, 2335 cancer-derived nonstop mutation extension (NSdb_MUT) with 2172 wildtype controls with the same extensions but starting with the wildtype STOP codon (NSdb_WT) plus 200 artificial sequences with one to ten copies of each of the 20 amino acids.

b, The verification of the inert sequence used as a filler to normalize the size of the library to 300 nt. No impact on eGFP expression due to the inert sequence was observed by flow cytometry of HEK293T cells transiently transfected with plasmids encoding the SMAD4 wildtype or *>C CDS with or without the inert sequence. Green bars depict cells expressing high eGFP and blue bars depict cells expressing low eGFP (i.e. expressing mCherry). n=4 independent biological replicates. Error bars depict mean ± SEM. The schematic of the plasmids was created in BioRender. Ghosh, A. (2024) BioRender.com/p44p477.

c, Dot plots representing the correlation between the read counts of the four biological replicates (BR) in the eGFP low, eGFP intermediate and eGFP high populations.

d, Flow chart depicting the analysis of the NGS reads, calculation of the median enrichment (M.E.) and the parameters defining the M.E. based categorization of the screening results into four groups. RPM- reads per million. The image was created in BioRender. Ghosh, A. (2024) BioRender.com/u61b559.

e, ROC (receiver operating characteristic) curve analysis comparing the median enrichment (M.E.) of the screen negative controls (the three stop codons and the 2172 NSdb_WT extensions) to the screen positive controls (25 degrons and six SMAD4 extension sequences) reveals a strongly significant area under the curve (AUC) of 0.96. The ROC curve analysis was performed in GraphPad Prism version 10.2.0 for MAC (GraphPad Software, Boston, Massachusetts USA, www.graphpad.com).

Figure S2

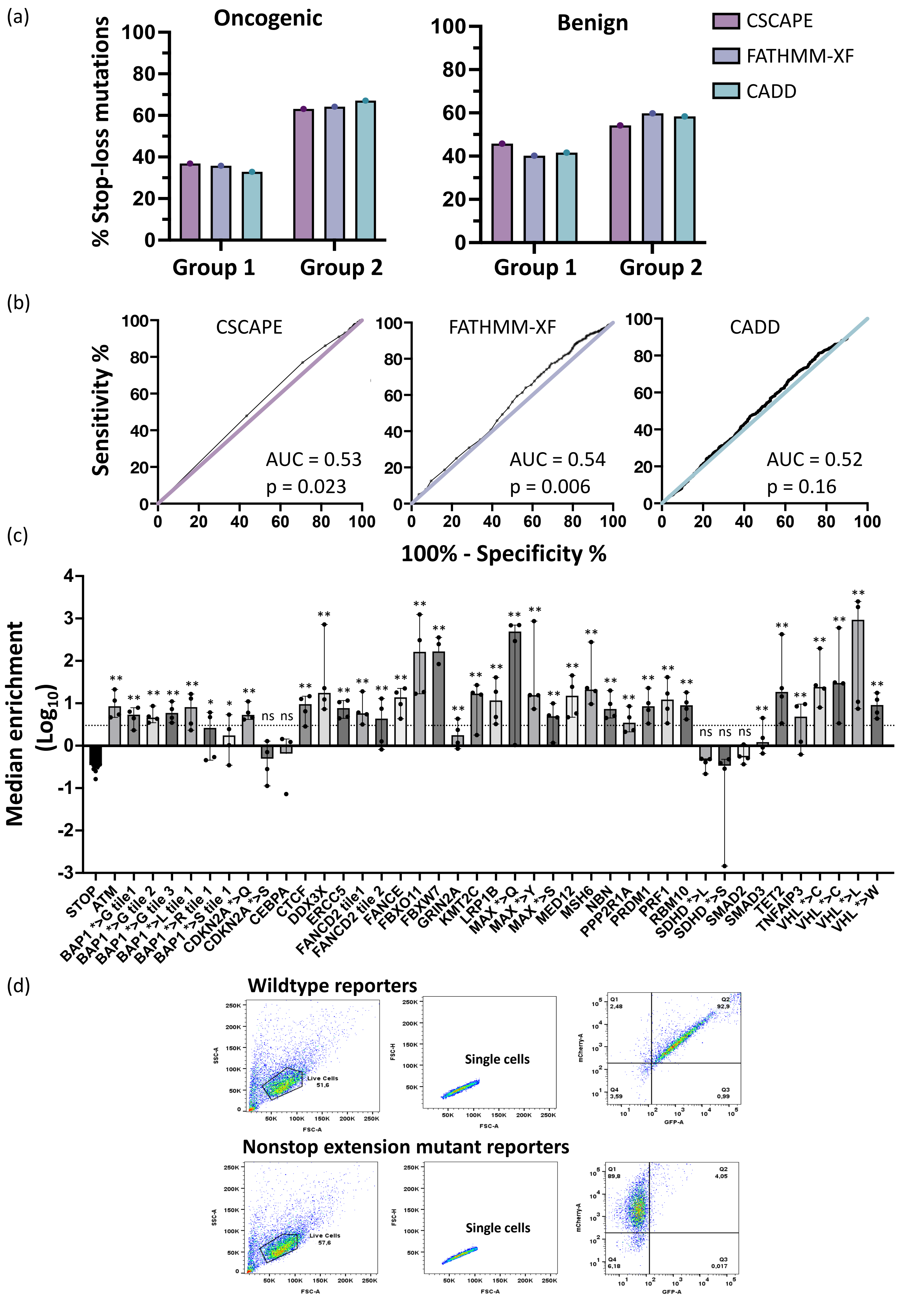
(a)
Oncogenic
Benign
% Stop-loss mutations
CSCAPE
FATHMM-XF
CADD
Group 1
Group 2
(b)
Sensitivity %
CSCAPE
AUC = 0.53
p = 0.023
FATHMM-XF
AUC = 0.54
p = 0.006
CADD
AUC = 0.52
p = 0.16
100% - Specificity %
(c)
Median enrichment (Log10)
STOP
ATM
BAP1 *>G tile1
BAP1 *>G tile 2
BAP1 *>G tile 3
BAP1 *>L tile 1
BAP1 *>R tile 1
BAP1 *>S tile 1
CDKN2A *>Q
CDKN2A *>S
CEBPA
CTCF
DDX3X
ERCC5
FANCD2 tile1
FANCD2 tile 2
FANCE
FBXO11
FBXW7
GRIN2A
KMT2C
LRP1B
MAX *>Q
MAX *>Y
MAX *>S
MED12
MSH6
NBN
PPP2R1A
PRDM1
PRF1
RBM10
SDHD *>L
SDHD *>S
SMAD2
SMAD3
TET2
TNFAIP3
VHL *>C
VHL *>C
VHL *>L
VHL *>W
(d)
Wildtype reporters
Live Cells 51,6
Single cells
Q1 2,48
Q2 92,9
Q3 0,99
Q4 3,59
Nonstop extension mutant reporters
Live Cells 57,6
Single cells
Q1 89,8
Q2 4,05
Q3 0,017
Q4 6,18
SSC-A
FSC-A
FSC-H
mCherry-A
GFP-A

Supplementary Figure S2: Prediction tools for analyzing the effect of nonstop extension mutations and tumor suppressor gene enrichments in the screen

a, Fraction of neutral (group 1) and effective (group 2) nonstop mutations scored as pathogenic or benign by three *in silico* prediction tools CSCAPE, FATHMM-XF and CADD.

b, ROC curve analysis comparing the group 1 extension mutations (neutral, as control) to the group 2 extension mutations (effective) for the prediction tools CSCAPE, FATHMM-XF and CADD. The ROC curve analysis was performed in GraphPad Prism version 10.2.0 for MAC (GraphPad Software, Boston, Massachusetts USA, www.graphpad.com).

c, Nearly all nonstop extension sequences from COSMIC Cancer Gene Census Tier 1 tumor suppressor genes in the NonStopDB showed a high median enrichment score highlighting their negative impact on protein expression. n=4 biologically independent replicates of the screen. Error bars depict median with 95% CI. *$P<0.05$, **$P<0.01$, two-tailed Mann-Whitney U test comparing each of the respective extensions independently to the three STOP controls.

d, Gating strategy used for flow cytometry. The live cells and singlets were selected from the total cell population. The eGFP-mCherry positive cells were subsequently gated using a quadrant gate. The gating strategy was used for all FACS experiments.

Figure S3

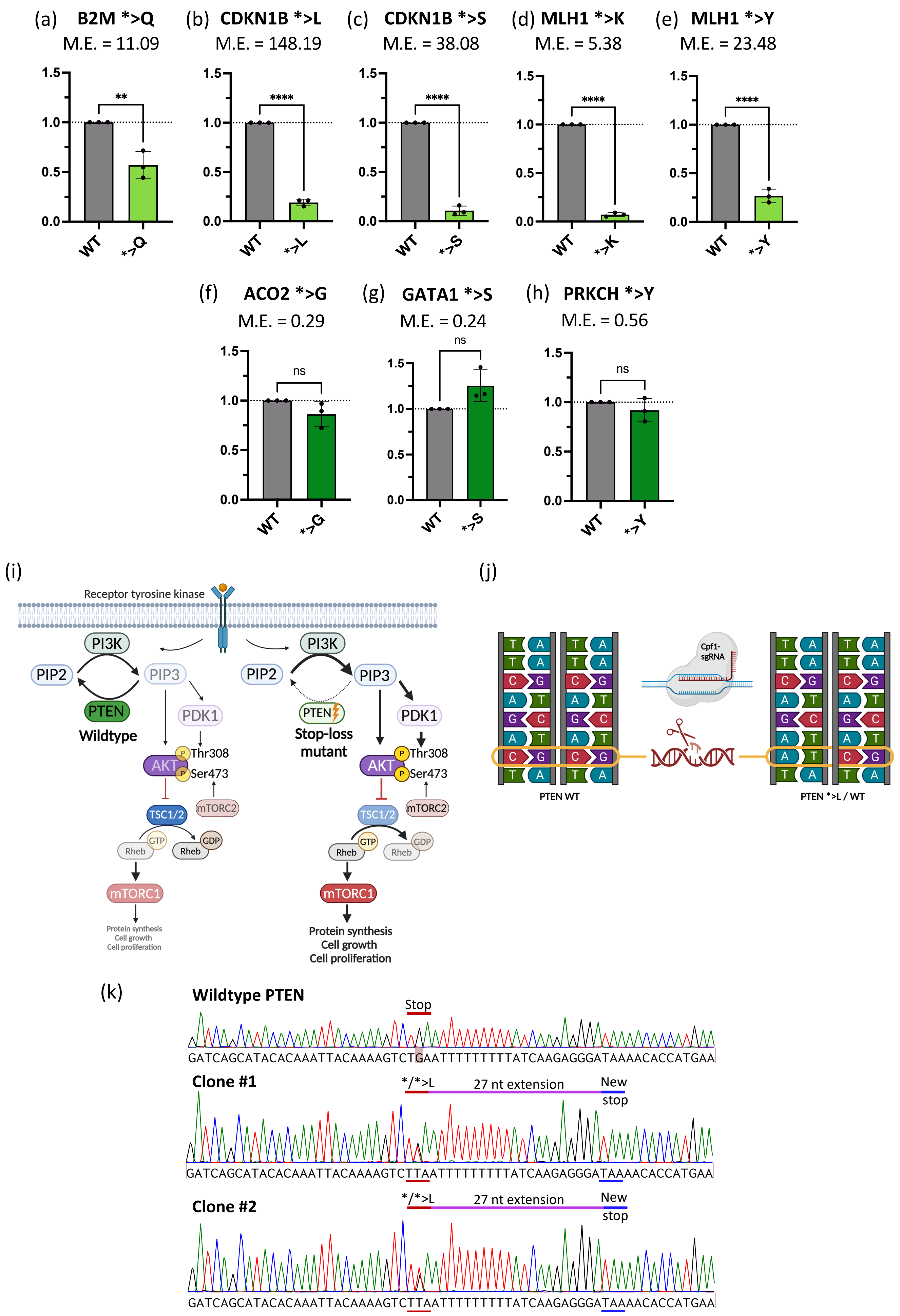

(a) B2M *>Q
M.E. = 11.09
(b) CDKN1B *>L
M.E. = 148.19
(c) CDKN1B *>S
M.E. = 38.08
(d) MLH1 *>K
M.E. = 5.38
(e) MLH1 *>Y
M.E. = 23.48
(f) ACO2 *>G
M.E. = 0.29
(g) GATA1 *>S
M.E. = 0.24
(h) PRKCH *>Y
M.E. = 0.56
(i)
Receptor tyrosine kinase
PI3K
PIP2
PIP3
PTEN
Wildtype
PDK1
AKT
Thr308
Ser473
TSC1/2
mTORC2
GTP
GDP
Rheb
mTORC1
Protein synthesis
Cell growth
Cell proliferation
Stop-loss mutant
(j)
Cpf1-sgRNA
PTEN WT
PTEN *>L / WT
(k)
Wildtype PTEN
Stop
GATCAGCATACACAAATTACAAAAGTCTGAATTTTTTTTTATCAAGAGGGATAAAACACCATGAA
Clone #1
/>L
27 nt extension
New stop
GATCAGCATACACAAATTACAAAAGTCTTAATTTTTTTTTATCAAGAGGGATAAAACACCATGAA
Clone #2
/>L
27 nt extension
New stop
GATCAGCATACACAAATTACAAAAGTCTTAATTTTTTTTTATCAAGAGGGATAAAACACCATGAA

Supplementary Figure S3: Validation of effective nonstop mutation-derived C-terminal extensions disrupting protein expression in overexpression and endogenous conditions

a-e, Effective nonstop extension mutations (group 2) fused to the HA-tagged CDS of the TSGs *B2M* (a), *CDKN1B* (b,c) and *MLH1* (d,e) lead to a loss of protein expression in western blot analysis in HEK293T cells after transient transfection. EV, empty vector; WT, wildtype. Relative protein expression was normalized to β-actin or Vinculin. n=3 independent biological replicates. Error bars depict mean ± S.D. **P<0.01, ***P<0.001, ****P<0.0001, unpaired t-test comparing the nonstop mutant extensions to the WT. Numerical source data and unprocessed blots are provided with the paper.

f-h, Neutral nonstop extension mutations (group 1) fused to the HA-tagged CDS of the genes *ACO2* (f), *GATA1* (g) and *PRKCH* (h) do not have a significant impact on protein expression in western blot analysis in HEK293T cells after transient transfection. EV, empty vector; WT, wildtype. Relative protein expression was normalized to β-actin or Vinculin. n=3 independent biological replicates. Error bars depict mean ± S.D. ns P>0.05, unpaired t-test comparing the nonstop mutant extensions to the WT. Numerical source data and unprocessed blots are provided with the paper.

i, Schematic depicting the impact of nonstop (stop-loss) mutation extended *PTEN* protein on the PI3K-mTOR signaling pathway.

j, Schematic depicting CRISPR precision genome editing to create the two heterozygous mutant HEK293T *PTEN* cell lines with the *>L mutation.

k, Sanger sequencing chromatograms depicting the heterozygous presence of the *>L (TTA) mutation in the two heterozygous mutant clonal cell lines in comparison to wildtype HEK293T cells.

The schematics in S3i,j were created in BioRender. Ghosh, A. (2024) BioRender.com/d74t858 and BioRender.com/x46u252, respectively. The chromatogram figure depicting the mutation in Fig. S3k was created using Benchling (www.benchling.com, accessed on 26 Feb 2024).

Figure S4

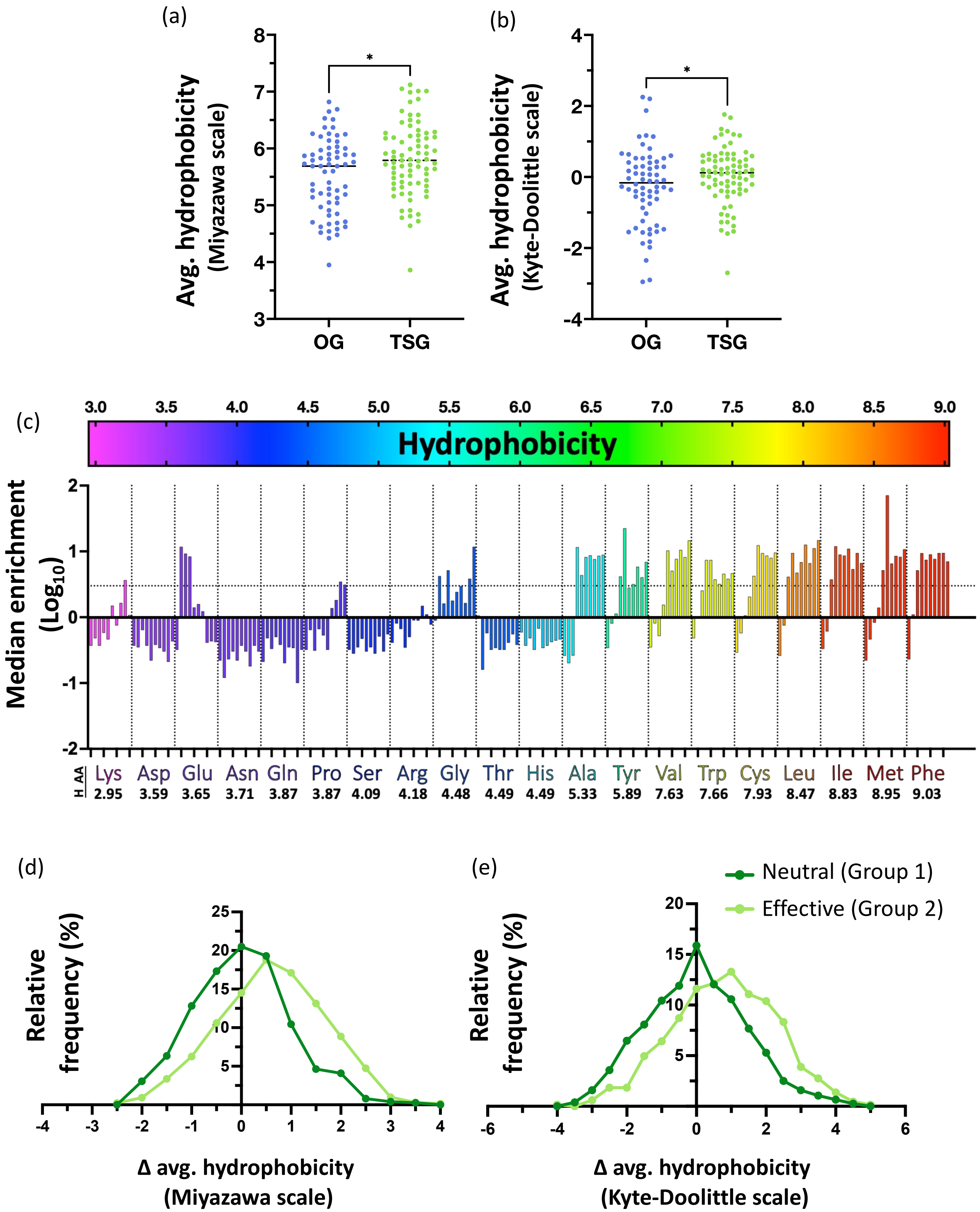

(a)
Avg. hydrophobicity (Miyazawa scale)
OG
TSG
*
(b)
Avg. hydrophobicity (Kyte-Doolittle scale)
OG
TSG
*
(c)
Hydrophobicity
Median enrichment (Log10)
H AA
Lys 2.95
Asp 3.59
Glu 3.65
Asn 3.71
Gln 3.87
Pro 3.87
Ser 4.09
Arg 4.18
Gly 4.48
Thr 4.49
His 4.49
Ala 5.33
Tyr 5.89
Val 7.63
Trp 7.66
Cys 7.93
Leu 8.47
Ile 8.83
Met 8.95
Phe 9.03
(d)
Relative frequency (%)
Δ avg. hydrophobicity (Miyazawa scale)
(e)
Neutral (Group 1)
Effective (Group 2)
Relative frequency (%)
Δ avg. hydrophobicity (Kyte-Doolittle scale)

Supplementary Figure S4: Hydrophobicity is an important determinant of nonstop extension-mediated loss of expression

a,b, C-terminal extensions occurring in tumor suppressor genes (TSG) possess a greater hydrophobicity than the extensions occurring in oncogenes (OG) using both Miyazawa (a) and Kyte-Doolitte (b) hydrophobicity scales. *$P<0.05$, two-tailed Mann-Whitney U test.

c, Extensions of one to ten times the same amino acid (bars from left to right for each amino acid) were tested in the screen and are depicted in the order of increasing hydrophobicity. The presence of multiple hydrophobic amino acid residues at the C-terminus of eGFP led to a loss of expression and a subsequent higher M.E. in the screen. Equivalent number of hydrophilic amino acids were less likely to elicit a loss of eGFP expression. Data depicted is the median of the four biological replicates of the screen. AA, amino acid; H, hydrophobicity Miyazawa scale.

d,e, At the individual gene level, the C-termini of effective C-terminal extensions are more hydrophobic in comparison to the wildtype protein coding CDS (represented as Δ avg. hydrophobicity, i.e. the difference in average hydrophobicity between the last 10 amino acids of the C-terminal extension and the last 10 amino acids of the CDS), than the C-termini of neutral extensions using both Miyazawa (d) and Kyte-Doolitte (e) hydrophobicity scales.

Figure S5

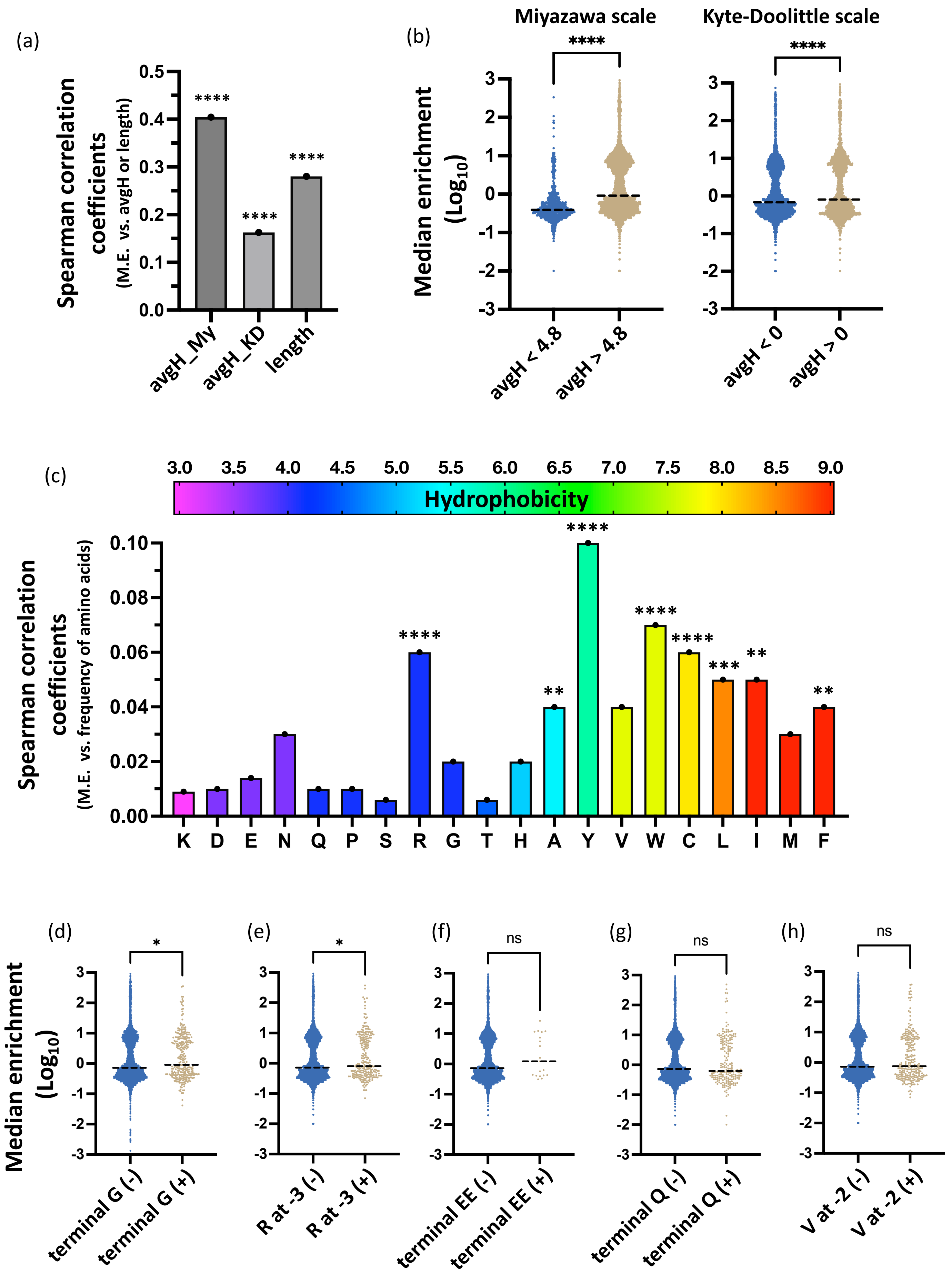

(a)
Spearman correlation coefficients
(M.E. vs. avgH or length)
avgH_My
avgH_KD
length
(b)
Miyazawa scale
Kyte-Doolittle scale
Median enrichment (Log_{10})
avgH < 4.8
avgH > 4.8
avgH < 0
avgH > 0
(c)
Hydrophobicity
Spearman correlation coefficients
(M.E. vs. frequency of amino acids)
K D E N Q P S R G T H A Y V W C L I M F
(d)
(e)
(f)
(g)
(h)
Median enrichment (Log_{10})
terminal G (-)
terminal G (+)
R at -3 (-)
R at -3 (+)
terminal EE (-)
terminal EE (+)
terminal Q (-)
terminal Q (+)
V at -2 (-)
V at -2 (+)
ns

Supplementary Figure S5: Parameters affecting nonstop extension-mediated loss of protein expression

a,b, Average hydrophobicity and length show a strong Spearman correlation to the median enrichment scores of the screen (a). For the whole library, extensions with a higher median enrichment have a higher average hydrophobicity using both Miyazawa (My) and Kyte-Doolitte (KD) hydrophobicity scales (b). ****P<0.0001, Spearman correlation coefficients.

c, The frequency of several amino acids including tyrosine, tryptophan, cysteine or arginine correlate with the suppressive effect of C-terminal extensions, but to a lesser extent than the average hydrophobicity and length as evidenced by the significant but weaker Spearman correlation coefficients. **P<0.01, ***P<0.001, ****P<0.0001, Spearman correlation coefficients.

d-h, Of the previously described degron motifs, a difference in M.E. scores for the entire library is only observed for C-terminal glycine and arginine at the minus three position, *P<0.05, Mann-Whitney U test. Terminal di-glutamic acid (EE), terminal glutamine (Q), and valine at the minus 2 position do not show significant differences in M.E. scores (f-h).
