# Supplementary material for: Suppressive cancer nonstop extension mutations increase C-terminal hydrophobicity and disrupt evolutionarily conserved amino acid patterns
Source: Nat Commun. 2024 Oct 25;15:9209. doi: 10.1038/s41467-024-52779-4 (PMC11502859; doi:10.1038/s41467-024-52779-4)
Supplement: Supplementary file 3 — Description of Additional Supplementary Files [file 41467_2024_52779_MOESM3_ESM.pdf]

<u>Description of Additional Supplementary Files</u>

Supplementary Data 1: Nonstop Extension Library & Screen Results.

This table lists all the nucleotide and amino acids sequences of the nonstop extensions (listed in the NonStopDB) along with the genes in which they occur, the NGS read counts of each extension from the high-throughput screen, the median enrichment of each extension as well as the M.E. group to which the extensions were assigned.

Supplementary Data 2: Tumor Suppressor Genes & Oncogenes.

This table lists the tumor suppressor genes (TSG) and oncogenes (OG) analyzed in the study. The classification of the genes as TSG or OG was obtained from the COSMIC Cancer Gene Census (CGC). The M.E. of the TSGs and OGs is additionally listed.

Supplementary Data 3: Oligonucleotide sequences.

This table lists all the oligonucleotide sequences used in this study (in the 5’ -3’ direction).
